# Numbers in the Blind's “Eye”

**DOI:** 10.1371/journal.pone.0006357

**Published:** 2009-07-23

**Authors:** Elena Salillas, Alessia Graná, Radouane El-Yagoubi, Carlo Semenza

**Affiliations:** 1 Department of Biology, University of Texas at San Antonio, San Antonio, Texas, United States of America; 2 Department of Psychology, University of Trieste, Trieste, Italy; 3 Department of Psychology, University of Provence, Provence, France; 4 Department of Neuroscience, University of Padova, Padova, Italy; 5 IRCCS Ospedale S. Camillo, Lido di Venezia, Italy; Macquarie University, Australia

## Abstract

**Background:**

Although lacking visual experience with numerosities, recent evidence shows that the blind perform similarly to sighted persons on numerical comparison or parity judgement tasks. In particular, on tasks presented in the auditory modality, the blind surprisingly show the same effect that appears in sighted persons, demonstrating that numbers are represented through a spatial code, i.e. the Spatial-Numerical Association of Response Codes (SNARC) effect. But, if this is the case, how is this numerical spatial representation processed in the brain of the blind?

**Principal Findings:**

Here we report that, although blind and sighted people have similarly organized numerical representations, the attentional shifts generated by numbers have different electrophysiological correlates (sensorial N100 in the sighted and cognitive P300 in the blind).

**Conclusions:**

These results highlight possible differences in the use of spatial representations acquired through modalities other than vision in the blind population.

## Introduction

Blind as well as sighted people show the SNARC effect[1]. This effect refers to the fact that, within a given interval, people in cultures where numbers are written from left to right are faster at making judgements (e.g. odd/even judgements) about smaller numbers with the left hand but are faster with their right hand for bigger numbers [2]. The SNARC effect has been interpreted to reflect the automatic activation of an internal representation of magnitude, where numbers are represented along a left-to-right oriented mental number line. Testing the number-space relationship in blindness entails a straightforward way of testing the suggested amodality of number semantics [see 3 for a review]. Finding that people deprived of visual world experience nonetheless show a spatial organization of number representation provides a clear indication of the existence of a modality-neutral, hardwired, core number representation. Number distance effects in the blind population provide converging evidence for this conclusion [4]. However, whether the blind process internal number representations the same way as sighted people remains to be shown. The present investigation aims at answering these questions.

Fischer, Castel, Dodd, & Pratt [5] investigated whether the internal representation of numbers could induce a shift of attention in the corresponding visual field. To address this question, they used a detection task in which irrelevant central cues (i.e., numbers 1, 2, 8 or 9) were presented followed by a lateralized target. A detection of the lateralized visual target was requested and detection times were measured. This way, congruent trials entailed targets on the right or left hemispace, preceded by large or small numbers, respectively, and incongruent trials entailed the opposite combination of number size and target location. Following large number cues (e.g., 8 or 9), detection times were faster for targets presented in the Right Visual Field (RVF), whereas after small numbers (e.g., 1 or 2) detection times were faster for targets presented in the Left Visual Field (LVF). In other words, Fischer et al. found a congruency effect with faster detection times for number size-location congruent trials. This finding suggests that the location of attention that follows number perception influences the location of attention in the visual field and that similar structures underlie attention shifts across internal spatial representations and external space. The electrophysiological correlates of this effect were recently described in sighted people [6]. In the present ERP study, we adapted this paradigm to the auditory modality in order to test blind individuals while measuring both behavioral detection times (behavioral experiment) and electrophysiological responses to the presentation of the target (ERP experiment).

People suffering congenital or early onset blindness have necessarily experienced numbers and numerosities in a different way compared to sighted people. We hypothesized that if shifts of attention induced by the perception of numbers differ between the blind and the sighted, then differences in the sensory (N100) and cognitive (P300) ERP components should be observed. Modulation of the sensory N100 has indeed been obtained with external cues in the blind (sound presented in the left or right auditory space [7]). In the current study, binaurally presented numbers were provided as cues in order to determine if the modulation of this component is also generated by an internal representation in the blind. Moreover, the serial unfolding of the auditory modality and the higher span of auditory working memory in the blind [8] could have an impact on the way that the blind manipulate numerical representations. Despite having similar left to right orientation in their numerical representation, and as a consequence of the kind of numerical input they receive, blind individuals may have a representation more dependent on working memory. The P300, which is an index of working memory load and has been interpreted as reflecting more controlled processes [9], [10], [21], [23] (i.e. whereby task relevant stimuli produce “matches” with internal representations or the maintenance of those representations in working memory), may be sensitive to number-space congruency in this auditory paradigm, especially in the blind who are required to compensate for lack of access to the visual modality. Hence, the development of representations with spatial characteristics through other modalities than vision, via intrinsic differences in processing, may have an impact on the general manipulation of these representations.

## Methods

### Participants

Seven sighted (mean age 34.4 years ranging from 22 to 50) and seven early-onset blind participants (mean age 35.4 years ranging from 25 to 50) completed the two experiments. All participants gave verbal informed consent to participate in the study, according to the rules enforced in the University of Trieste. Furthermore, for the blind group, this informed consent was obtained after a talk at the Blind Italian Union, Trieste provincial section. In this talk, the experimenter gave some details of the ERP technique, data collection, and the task to be performed, and participants willing to collaborate were recruited. No other revisions are mandatory in Italy for the methodology used in this study.

### Stimuli

Numbers were recorded by a female speaker and compressed to a fixed duration of 350 ms. using Wavelab 4.0. Auditory intensity of large (8 and 9) and small (1 and 2) numbers was equivalent (70 dB.). Lateralized auditory targets were presented using dichotic listening: a target sine wave sound (166.67 Hz.) was presented in one ear, and pink noise was presented in the other ear. Therefore a target on the right occurred when the sine sound was presented in the right ear and pink noise was presented in the left ear and vice-versa for targets on the left. Both right and left targets were identical and were generated by cross-splicing the same sound to the corresponding channel, therefore the two targets had exactly the same acoustic characteristics. For catch-trial stimuli, pink noise was presented in both channels. All experimental and catch sounds had a duration of 100 ms.

### Procedure

All participants did a behavioural experiment, where detection times after the presentation of the target were collected, and a second experiment, where ERPs to the presentation of the target were measured. These two separate experiments made it possible to obtain reaction times immediately after the presentation of the target, while avoiding the ERP response contamination with response preparation.

**Figure 1:**
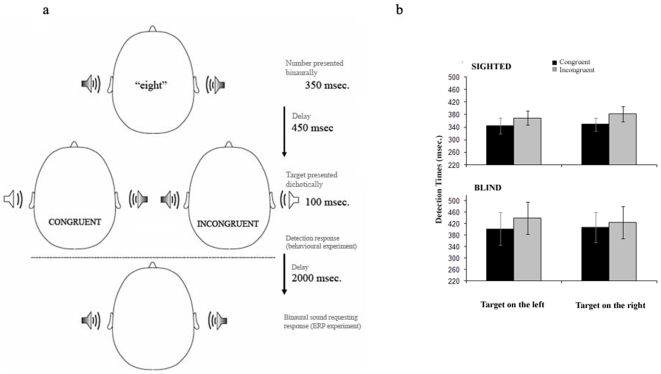
a. Sequence of the stimulus in the two experiments. Detection responses were requested right after the target in the behavioural experiment and after the second sound in the ERP experiment. b. Behavioral results. Mean detection times plotted with standard error of the mean. A main effect of congruency can be observed, without interaction with group or with side of presentation of the target.

Our paradigm (fig. 1a) consisted of the binaural auditory presentation of large (8 or 9) or small (1 or 2) numbers. The number was followed by a fixed delay of 450 ms., and then a lateralized target was presented through dichotic listening. This delay showed the strongest effect in the study of Fischer and collaborators [5]. The experimental session was divided into 4 blocks of 60 trials, during which a total of 240 trials were presented. ERPs were computed by averaging the EEG recordings associated with the presentation of the target.

Auditory stimuli were presented through headphones with a fixed volume for all participants (audio format PCM, 44100 Hz, 16 bits, stereo). A number (1, 2, 8 or 9) was binaurally presented with a duration of 350 ms. After a fixed delay of 450 ms. a target was presented through dichotic listening, or a catch-noise sound was presented binaurally. Both target sounds and catch sounds had a duration of 100 ms. In the behavioural experiment, detection of the target was requested by immediately pressing a button. If a catch sound had been presented, the participant was instructed to do nothing. In the ERP experiment, after a delay of 2000 ms. from the onset of the target, a different sound signalled the moment in which the subject had to press a button if a target had appeared before (delayed detection task). ERPs were measured from the initiation of the target or catch sounds. In both experiments, the hand used for the response was intermixed across blocks and varied across participants. All participants were asked to close their eyes while performing the tasks.

### EEG Recording and data analysis

Continuous EEG was recorded from 28 scalp electrodes mounted in an elastic cap (Electro-Cap international) and located at standard left and right hemisphere positions over frontal, central, parietal, occipital and temporal areas (International 10/20 System, at Fz, Cz, Pz, Oz, Fp1, Fp2, F3, F4, C3, C4, P3, P4, O1, O2, F7, F8, T3, T4, T5, T6, Ft7, Ft8, Fc3, Fc4, Cp3, Cp4, Tp7, Tp8). These recording sites, plus an electrode placed over the right mastoid, were referenced to the left mastoid electrode online. The data were recorded continuously throughout the task by a SynAmps amplifier and NeuroScan 4.3 software. Each electrode was re-referenced off-line to the algebraic average of the left and right mastoids. Impedances of these electrodes never exceeded 5 kΩ. The horizontal electro-oculogram (HEOG) was recorded from a bipolar montage with electrodes placed 1 cm. to the left and right of the external canthi; the vertical (VEOG) was recorded from a bipolar montage with electrodes placed above and below the right eye, to detect eye movements. EOG activity were detected by wavelet analysis and corrected using a regression method in the time domain [11]. Epochs from 100 ms. before and 600 ms. after the presentation of the target were extracted from the EEG. The EEG and EOG were amplified by a Synamp's amplifier digitized at a rate of 500 Hz and filtered with a band pass of 0.01–30 Hz. Another filtering (low-pass filtering cutoff of 5 Hz. [12] was performed in order to remove alpha rhythm that could be different between blind and sighted participants, with eyes closed. Epochs were excluded from averaging if they contained amplitudes outside the range +/−150 µV at any EEG site. ERPs were extracted by averaging trials separately for subjects, electrodes, and experimental conditions.

The 100 ms. period preceding the target was used as the prestimulus baseline. ERP averages were analysed by computing the mean amplitude in selected latency windows. ANOVAs were used for all statistical tests and were carried out with the Greenhouse-Geisser correction for non-sphericity [13]. To explore the potential topographic differences, ANOVAs were conducted separately for midline and lateral electrodes. ANOVAs for midline electrodes had a repeated-measures design, with group (blind/ighted) as a between-subjects factor, and congruent/incongruent, side of presentation of the target (Left Visual Field (LVF)/Right Visual Field (RVF)), Localization (2 Regions Of Interest [ROIs] or Area; Anterior and Posterior) and electrodes (2 for each ROI with Anterior including: Fz, Cz, and Posterior including: Pz and Oz) as within-subjects factors. ANOVAs for lateral electrodes also had a repeated-measures design with congruency (congruent/incongruent), side of presentation of the target (Left Visual Field (LVF)/Right Visual Field (RVF)), hemispheres (Left vs. Right), Localization (2 Regions Of Interest [ROIs] or Area; Anterior, and Posterior), and electrodes (6 for each ROI with Left Anterior including: FP1, F7, F3, FT7, FC3, C3; Left Posterior: CP3, T3, TP7, P3, T5, O1; Right Anterior: FP2, F8, F4, FT8, FC4, C4; and Right Posterior: CP4, T4, TP8, P4, T6, O2). A similar ANOVA was performed for the catch trials with Number (large and small) as within-subjects factor and group as a between-subjects factor with all the rest of factors being the same: ROI/hemisphere/electrode.

## Results

### Behavioral results

We determined the presence of a congruency effect for both groups in terms of their reaction times (RTs, fig. 1b). This experiment was designed in order to provide reaction times, as the delayed-detection task in the ERP experiment could obscure any behavioural effects due to the interval between the target and response. A three factorial ANOVA with 2 (congruency) ×2 (side) as repeated measures, and 2 (group) as a between-subjects factor showed an effect of congruency (F(1,12) = 17.55, p = 0.001; Congruent trials: Mean = 376.12 ms., SD = 29.78 ms; Incongruent trials: Mean = 403.9 ms., SD = 29.95 ms.) but no interaction with side (F(1,12) = 0.3, p = 0.58) or group (F(1,12) = 0.01, p = 0.92). The main effect of group was not significant (F = 0.9, p = 0.36)^1^ That is, regardless of side or group, targets in congruent trials were detected faster than targets in incongruent trials.

### ERP results

**Figure 2:**
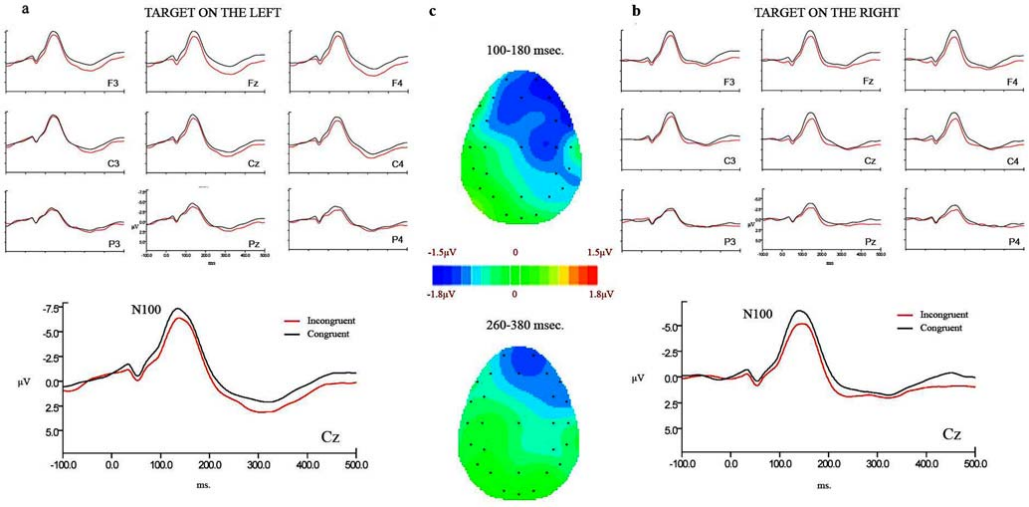
ERPs elicited by the target in sighted participants. a) target on the left; b) target on the right. Black line represents congruent trials and red line incongruent trials. c) Difference between congruent and incongruent conditions in the two latency windows.

**Figure 3:**
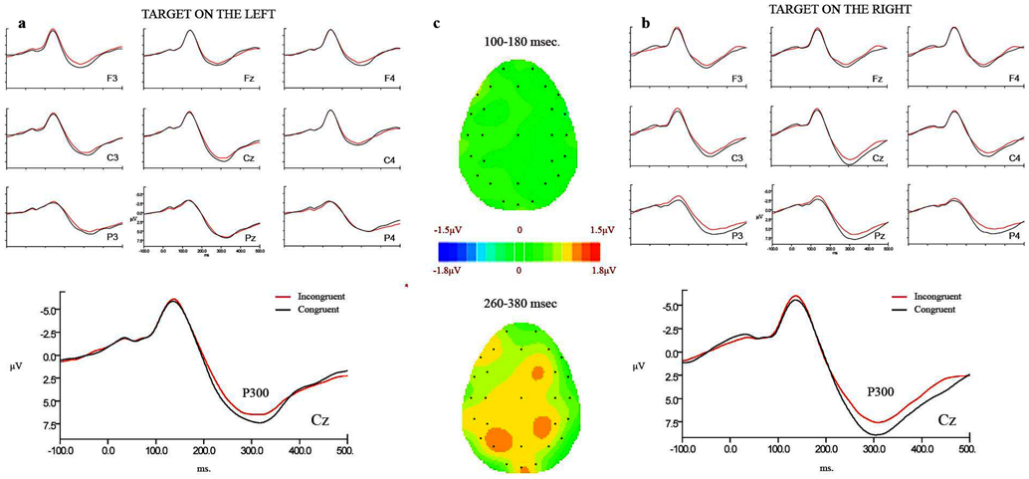
ERPs elicited by the target in blind participants. a) target on the left; b) target on the right. Black line represents congruent trials and red line incongruent trials. c) Difference between congruent and incongruent conditions in the two latency windows. A continuation of the congruency effects on the N100 is shown in the latency window of the P300: same scalp distribution as for the effect on the negativity can be seen.

In order to capture the stimulus processing phase and to separate it from the motor preparation of the response, a delayed response paradigm was used (see Fig. 1a) in the ERP experiment. Figures 2 and 3 provide a comparison of the ERPs for congruent vs. incongruent trials on each side of the presentation of the target for sighted (Fig. 2) and blind (Fig. 3) participants. A visual inspection of the ERPs of congruent vs. incongruent trials showed two main components that were differentially modulated by congruency depending on the group: while a negativity for the latency and distribution of the N100 component showed a modulation by congruency in sighted individuals, a positivity at the latency and centro-parietal distribution of the P300 component showed modulation by congruency in blind individuals. A peak-latency analysis of a window between 80 and 180 ms. for the N100 and between 200 and 500 ms. for the P300 did not reveal any difference in latency between the groups, or across conditions for any of the components in the experimental trials (average peak latency of 140.5 ms. for the N100 and 314.2 for the P300).

Statistical analysis of mean amplitudes by a 2 (congruency) ×2 (side) ×2 (hemisphere) ×2 (ROI: anterior/posterior) ×6 (electrode) ANOVA for the lateral electrodes and by a 2 (congruency) ×2 (side) ×2 (ROI: anterior/posterior) ×2 (electrode) ANOVA for the midline electrodes confirmed these observations. The main effect of group was not significant (lateral: F(1,12) = 0.147, p = 0.7; midline: F(1,12) = 0.54, p = 0.47). An interaction between group and congruency showed that the N100 (latency band between 100 and 180 ms.) was modulated by congruency only in sighted participants (lateral: F(1,12) = 8.50, p = 0.013; midline: F(1,12) = 8.33, p = 0.014). In this group, congruent trials elicited larger amplitudes than incongruent trials, independent of the location of the target (lateral: F(1,6) = 10.02, p = 0.019; midline: F(1,6) = 7.9, p = 0.03). The N100 for both groups, as well as the congruency effect in sighted participants, was localized to anterior sites as shown by a main effect of ROI (lateral: F(1,12) = 39.5, p<0.001; midline: F(1,12) = 75.08, p<0.001) and the interaction between congruency and ROI in the sighted participants (lateral: F(1,6) = 8.08, p = 0.03; midline: F(1,6) = 10.39, p = 0.018; effect of congruency in anterior sites: lateral: F(1,6) = 15.1, p = 0.008; midline: F(1,6) = 13.04, p = 0.01; posterior sites: n.s.). The P300 (latency band between 260 and 380 ms.) also showed a modulation by congruency, but in this case only for the blind group, as shown by the group × congruency interaction (lateral: F(1,12) = 12.31, p = 0.04; midline: F(1,12) = 10.38, p = 0.007). Larger amplitudes for congruent trials appeared in the blind group, with no interaction with the side of presentation of the target (lateral: F(1,6) = 6.38, p = 0.04; midline: F(1,6) = 11.39, p = 0.015)^2^. A main effect of group appeared, with generally larger amplitudes for the blind than for the sighted participants (lateral: F(1,12) = 4.25, p = 0.06; midline: F(1,12) = 11.97, p = 0.005).

### Catch trials

In the latency band of the N100 an effect of ROI was also shown in the catch trials (F(1,12) = 7.2, p = 0.02) for lateral electrodes and in midline electrodes (F(1,12) = 16.9, p = 0.001), therefore, the distribution of this component was the same for experimental and catch trials, with larger amplitudes in anterior sites. A number × hemisphere interaction was also present (F(1,12) = 6.31, p = 0.02). This interaction showed larger amplitudes for small numbers in the right hemisphere, while there were no differences in the left hemisphere. The simple effects for this interaction were not statistically significant. No interactions by group or other effects were found for this component.

In the latency band of the P300 (300–420 ms.) a number × hemisphere interaction was found in the lateral electrodes (F(1,12) = 17.9, p = 0.001). Large numbers elicited a greater positivity than small numbers in the right hemisphere. The simple effects for this interaction did not reach significance.

## Discussion

Our results are consistent with the behavioural findings from Fischer et al. [5] and the ERP data from Salillas et al. [6], both obtained in the visual modality, and extend them to the auditory modality. Our behavioural data show a similar overall organization of the mental number line for sighted as well as blind individuals, as previously demonstrated by Castronovo and Seron [1]. Importantly, the present work shows that the size of a spoken number generates shifts of spatial attention in the auditory space in both groups. However, congruency had a different effect on the ERPs for blind and sighted individuals.

The amplitude of the early sensory N100 component was modulated by congruency only in the *sighted group*. Previous work has shown enhanced N100 amplitude for the same auditory stimulus when presented in an attended versus unattended location [14], [15], [16]. It has been suggested that these effects are generated by an enhancement of information received from the selected source, according to the amount of attention allocated to that input [16]. Evidence further suggests that effects of selective attention to location exert an early influence in the primary auditory cortex [17], [18]. In our experiment, access to a spatially-organized internal numerical representation [19] exerts spatial shifts of attention over auditory space. Moreover, as signalled by the modulation of the N100 for the sighted participants, the effect of congruency between number and target location can be explained as an amplification of the auditory sensory processes. Importantly, this amplification may be the consequence of a top down mechanism: the sensorial activity of primary areas seems to be modulated by a higher order representation.

By contrast, in the *blind group*, the ERP congruency effect was only observed in the cognitive P300 and not in the early N100 component. Moreover, this component showed larger amplitudes for this group. The P300 effect is typically attributed to the increase of relevance of the cued location [20], [21]. It is also described as reflecting higher cognitive processes of attention allocation, retrieval and maintenance of a representation in working memory [10], [14]. Attending to these functional explanations of P300 the larger P300 amplitude found for congruent trials signals that a trace of the relevance of a location may have been held in working memory. The absence of the same modulation in the N100 in the blind suggests that the activation of the number representation does not influence the sensory processing of the target for this group. Accordingly, blind individuals may have restricted processing of congruency to a cognitive level (P300), applying working memory resources to the computation of congruency. The absence of a modulation of P300 amplitude by congruency in the sighted group could be due to the modality of presentation of the stimuli. Provided that the visual modality is functional, auditory working memory in the sighted is less necessary than in the blind [22]. In other words, a different use of spatial representations may derive from the lack of vision. The manipulation of representations like those of numbers may become more dependent on working memory resources and thus more controlled [23], its impact on attention remaining at a higher level.

In summary, although our RT data as well as those of previous behavioural studies [1], [4] show the same pattern for blind and sighted participants, our study has uncovered different neurophysiological correlates for number manipulation in the two groups and, therefore, different underlying processes. The absence of visual input and the use of the auditory modality with less discriminative power and greater working memory requirements, may lead blind people to manipulate the mental number line in a more controlled way than sighted people, relying on working memory while showing no effects at the sensory level. In other words, receiving the numerical input through the auditory or tactile modalities may have generated a representation ultimately linked to working memory in its manipulation. This could explain why superior number estimation performance is found compared to sighted people [24]. To rely on a more controlled process when doing number estimation would result in a more accurate outcome. Similarly, the SNARC effect has been found when a manipulation of the mental number line becomes more controlled as in numeric comparison tasks [25], [26].

In conclusion, this study demonstrated how numbers represented in the blind mind's “eye” are processed in a more controlled way compared to sighted people, which may explain the superior performance of non-sighted people in some estimation tasks.

## 

### Footnotes

1. One of the blind participants was very slow in the behavioural experiment, which explains the apparent longer reaction times for this group. This participant was not excluded from the analysis in order to have the same individuals for both experiments. The analysis for the behavioural data without this participant showed the same main effect of congruency (F(1,11) = 14.33, p = 0.003) with no interaction with group or side of the target and with mean RTs of 373.28 ms. for the blind group and of 361.73 for the sighted group.

2. An effect of congruency was detected in the sighted group for the P300 window in the lateral analysis (F(1,6) = 6.83, 0.04; F(1,6) = 3.06, p = 0.1) i.e. bigger amplitude for incongruent than for congruent trial. This effect had the same anterior distribution of the N100 for this group, which clearly indicates contamination from the previous component: differing from the broadly distributed P300 congruency effect for the blind group, a congruency × ROI interaction was observed for the sighted group (F(1,6) = 6.01, p = 0.05; F(1,6) = 7.76, p = 0.03), the same interaction that was found for the N100 in this group. If a P300 modulation with bigger amplitudes in congruent trials should be found for the sighted group this would have appeared in at least some posterior sites, which was not the case. Therefore, the bigger negativities for congruent trials in the anterior sites within the P300 latency band for the sighted group suggest a contamination from the earlier N100 effect.
